# Impact of COVID-19 on Diaphragmatic Function: Understanding Multiorgan Involvement and Long-Term Consequences

**DOI:** 10.3390/jcm13216493

**Published:** 2024-10-29

**Authors:** Katarzyna Anna Pietranis, Amanda Maria Kostro, Zofia Dzięcioł-Anikiej, Diana Moskal-Jasińska, Anna Kuryliszyn-Moskal

**Affiliations:** 1 Department of Rehabilitation, Medical University of Bialystok, 24A M. Skłodowskiej-Curie St., 15-276 Bialystok, Poland; amanda.kostro@umb.edu.pl (A.M.K.); zofia.dzieciol-anikiej@umb.edu.pl (Z.D.-A.); anna.kuryliszyn-moskal@umb.edu.pl (A.K.-M.); 2 Department of Clinical Phonoaudiology and Speech Therapy, Medical University of Bialystok, 37 Szpitalna St., 15-295 Bialystok, Poland; diana.moskal-jasinska@umb.edu.pl

**Keywords:** COVID-19, long-COVID, diaphragm, respiratory dysfunctions, diaphragmatic disorders

## Abstract

The COVID-19 pandemic has brought significant attention to the respiratory system, with much focus on lung-related disorders. However, the diaphragm, a crucial component of respiratory physiology, has not been adequately studied, especially in the context of long COVID. This review explores the multipotential role of the diaphragm in both respiratory health and disease, emphasizing its involvement in long-term complications following SARS-CoV-2 infection. The diaphragm’s fundamental role in respiratory physiology and its impact on balance and posture control, breathing patterns, and autonomic nervous system regulation are discussed. This review examines complications arising from COVID-19, highlighting the diaphragm’s involvement in neurological, musculoskeletal, and inflammatory responses. Particular attention is given to the neuroinvasive impact of SARS-CoV-2, the inflammatory response, and the direct viral effects on the diaphragm. The diaphragm’s role in long COVID is explored, with a focus on specific symptoms such as voice disorders, pelvic floor dysfunction, and sleep disturbances. Diagnostic challenges, current methods for assessing diaphragmatic dysfunction, and the complexities of differentiating it from other conditions are also explored. This article is the first to comprehensively address diaphragmatic dysfunction resulting from COVID-19 and long COVID across various physiological and pathological aspects, offering a new perspective on its diagnosis and treatment within a multisystem context.

## 1. Introduction

Since the onset of SARS-CoV-2 infections approximately four years ago, a significant part of the population has recovered from COVID-19, with the severity of the illness varying among individuals. Following infection, many individuals have experienced weakened respiratory muscles, restricted lung ventilation, and significantly impaired reserve function [1,2]. Additionally, some individuals have developed long-term effects from the disease, commonly referred to as ‘long COVID’ or ‘post-COVID syndrome’. However, the exclusivity, duration, and pathophysiology of these abnormalities, particularly among patients who have undergone invasive mechanical ventilation, are still being studied. Mechanical ventilation frequently leads to diaphragm inactivity due to factors such as inappropriate pressure support, complications from critical illness polyneuropathy, patient-ventilator asynchrony, and widespread infection. This inactivity can lead to myofiber atrophy and diminished diaphragm contraction, resulting in ventilator-induced diaphragmatic dysfunction [3]. Recent observations have highlighted that mechanically ventilated patients with COVID-19 face a significant risk of developing pneumomediastinum [4,5]. Extensive changes in the tracheal and major airway cartilage, characterized by fibrous-hyaline degeneration and unusual regenerative processes involving cartilage tissue remodeling, are distinctive markers directly linked to COVID-19 [4]. Scientists confirm that symptoms such as cough, sputum production, and shortness of breath can persist for an extended period, even after the infection has subsided in COVID-19 patients. Notably, dyspnea, especially upon exertion, continues to affect a considerable number of survivors, even those with normal lung and heart function [6,7]. This prompts the search for other possible causes of dyspnea, including those related to diaphragmatic dysfunction. Additionally, it underscores the long-term impact of the virus and highlights the need for ongoing monitoring and treatment strategies to address these persistent effects [8].

In clinical practice, the importance of the respiratory diaphragm is gaining increasing recognition, particularly in its association with general body weakening, which is especially critical in patients in serious conditions. Diaphragm weakness in critically ill patients contributes to the difficulty of weaning from mechanical ventilation due to the diaphragm’s inability to sustain the necessary workload for independent breathing. Failure to wean can result from diaphragm wasting caused by inactivity, contractile weakness, and/or insufficient signaling from the central nervous system to the diaphragm [9,10,11,12].

Notable changes in diaphragmatic function have been observed in patients with systemic lupus erythematosus, where a link to reduced functional capacity has been identified [13]. Additionally, a relationship between neuromotor weakness and diaphragm contractility has been found in post-stroke patients with hemiplegia [14,15]. Diaphragm dysfunction is also present in conditions like Duchenne muscular dystrophy [16,17].

In chronic obstructive pulmonary disease (COPD), respiratory muscle dysfunction is a key feature of both acute and chronic respiratory failure. The diaphragm and accessory inspiratory muscles are subjected to increased load due to greater lung resistance and elastance, along with higher ventilatory demands [18]. Reduced inspiratory muscle strength has been directly linked to a diminished ability to maintain balance [14,15].

In patients with unilateral cervical radiculopathy (CR), a study revealed a dysfunctional breathing pattern during both normal and deep breathing, along with a unilateral reduction in diaphragmatic excursion on the side affected by radiculopathy, compared to the control group [19].

In this article, we summarize the recent literature on diaphragmatic dysfunctions caused by COVID-19, complications related to the diaphragm and the potential involvement of the diaphragm in long COVID. This article is the first to detail diaphragmatic dysfunction in multiple aspects resulting from COVID-19 and long COVID.

## 2. The Multipotential Role of the Diaphragm in Respiratory Physiology and Pathological Conditions

**Figure 1:**
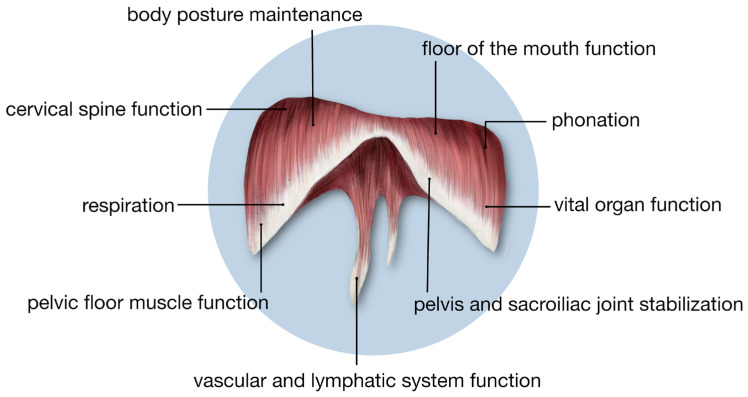
The physiological impact of the diaphragm on the human body.

The diaphragm is the most important respiratory muscle and is involved in several physiological processes, including coughing, sneezing, swallowing, vocalization, defecation, and urination [20,21]. The physiological and anatomical connections of the diaphragm throughout the body are shown in Figure 1 [22,23,24]. The diaphragm is a key postural muscle, primarily responsible for maintaining balance, lumbar stability, and effective neural coordination [25,26]. The significance of the diaphragm extends beyond respiration to include proprioception, pain perception, and emotional states. Furthermore, the functioning of this muscle is essential for the lymphatic and circulatory systems [27,28]. Breathing facilitated by the diaphragm positively impacts cognitive functions by stimulating synaptogenesis and the formation of working memory. Proper nasal breathing activates brain areas responsible for attention, sensory processing, concentration, and emotions, while also stimulating the cerebellum, which is responsible for spatial thinking, motor precision, and balance [29]. Maintaining balance is possible through the cooperation of the following systems: vestibular, visual, tactile, proprioceptive, and auditory [30].

### 2.1. Balance and Posture Control

The diaphragm is an important part of the system responsible for balance. Its respiratory function supports the cardiovascular and lymphatic systems and ensures the stability of the lumbar spine by regulating pressure in the abdominal cavity. Studies indicate that following thoracic surgery, diaphragm function impairment is accompanied by a deterioration in balance parameters [31,32,33]. The influence of the diaphragm on maintaining balance can be explained by its anatomical location and function—it stabilizes the lumbar spine. There are several possible explanations for this phenomenon, including afferent vagal conduction and the activation of the vestibulo-autonomic reflex, the presence of specialized mechanoreceptors (proprioceptors) in the frontal pad of the diaphragm and their stimulation by intra-abdominal pressure, and the fatigue of the diaphragm and phrenic nerve paralysis. When breathing efficiency is impaired and the diaphragm’s zone of apposition is reduced, deep sensory receptors may function incorrectly, leading to the compromised diaphragmatic control of body posture [31].

### 2.2. The Pivotal Role of the Diaphragm in Breathing Patterns

Diaphragmatic breathing is a part of feedback that improves vagal tone by stimulating the relaxation response of the parasympathetic nervous system. In turn, changing the breathing pattern from lower rib to thoracic breathing leads to the overactivity of the sympathetic nervous system and the hyperactivity of the diaphragm. A reduction in the diaphragm’s zone of apposition adopts an inspiratory pattern. If the diaphragm’s resting position is lowered, the lumbar muscles become hyperactive, resulting in increased lumbar lordosis [34,35].

The restriction of diaphragm movement results in the inadequate modulation of intra-abdominal pressure [31]. Research by Holt et al. demonstrated that, in addition to muscle spindles, pressure-sensitive mechanoreceptors are also present in the diaphragm. The insufficient modulation of intra-abdominal pressure may lead to the weak stimulation of these proprioceptors, thereby disrupting the flow of proprioceptive information to the central nervous system [36]. Diaphragmatic fatigue may result from either contractile dysfunction or a lack of neuronal activation, referred to as peripheral and central fatigue, respectively [31,37].

### 2.3. Innervation of the Diaphragm—The Role of the Phrenic Nerve and Autonomic System

When considering the role of the phrenic nerve, it is important to note that this structure is responsible for the sensory (proprioceptive) innervation of the diaphragm. The phrenic motor units are involved in more than just controlling breathing; they also contribute to non-respiratory functions such as swallowing, vocalization, and clearing waste from the airways through expectoration [38]. The right dome of the diaphragm is approximately 1.9 cm higher than the left. However, at the end of a deep inhalation, this difference decreases to about 1.5 cm. The right phrenic nerve conducts electrical impulses at a faster velocity than the longer left phrenic nerve [37,38,39,40]. It can be assumed that if the position of the diaphragm is not physiological, the phrenic nerve, when irritated, will send abnormal proprioceptive signals to the central nervous system. The precise physiological impact of activating sensory afferent fibers in the diaphragm via the phrenic nerve is challenging due to the diversity of afferent fiber types [41]. Diaphragmatic afferent neurons can initiate various physiological responses such as increased sympathetic outflow, higher arterial blood pressure, improved ventilation, and reduced intercostal motor output [42,43]. These responses vary depending on the receptor that is activated and the specific context of stimulation. Additionally, these neurons can activate cortical somatosensory neurons, influencing the perception of breathing and emotional responses to respiratory stress, which is particularly relevant for understanding dyspnea [44,45,46].

In the entire respiratory system and the sequence of brain area activations, it is also important to mention the vagus nerve. The involvement of the vagus nerve is crucial for coordinating the diaphragmatic esophageal sphincter area to facilitate the passage of a food bolus [47,48].

It is reasonable to state that the diaphragm plays a crucial role in maintaining static balance, making the balance function inextricably linked to breathing. However, it is also important to consider the sympathetic nervous system, which is closely connected with the stellate ganglion and the subdiaphragmatic and diaphragmatic ganglia throughout this process. This is evidenced by the negative impact of breathing disorders on the sympathetic nervous system, where long-term, excessive activation can lead to chronic stress, increased muscle tension, and other related disorders [49,50,51].

## 3. Complications After COVID-19 in the Context of Diaphragm Involvement

**Figure 2:**
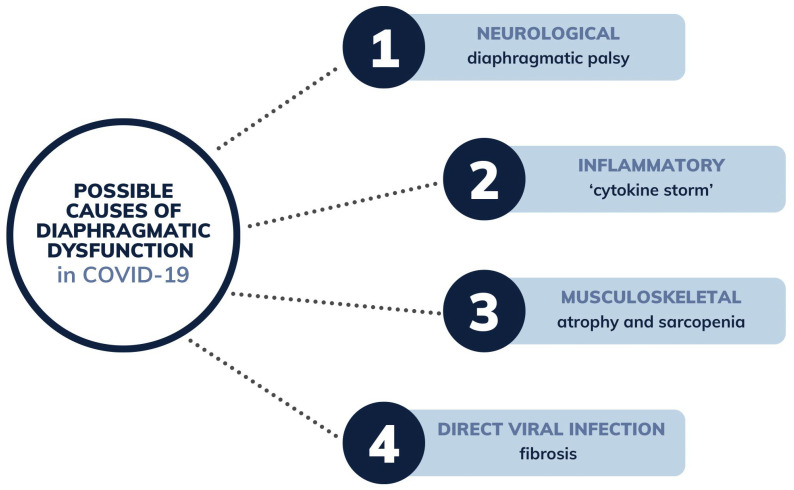
Possible main causes of diaphragmatic dysfunction resulting from COVID-19.

COVID-19 is an intensely contagious illness that primarily affects the respiratory system, precipitating significant physical and psychological impairments. Common symptoms of the disease include fever, fatigue, and persistent coughing. Additionally, patients often report muscle soreness, dyspnea, nausea, vomiting, diarrhea, chest tightness, and headaches [52,53]. The majority of SARS-CoV-2 infections are either asymptomatic or display minimal symptoms that do not necessitate hospital care. However, the escalation of symptoms signals the transition to more severe stages that require hospitalization for interventions such as thromboembolism prevention, oxygen therapy, and rehabilitative care. Despite therapeutic interventions, some instances may advance to acute respiratory distress syndrome (ARDS), which complicates recovery by inducing respiratory muscle weakening, secondary pulmonary fibrosis, and impaired diffusion [54,55]. In the context of diaphragmatic disorders resulting from COVID-19, there are multiple possible causes, some of which are illustrated in the diagram [Figure 2].

### 3.1. Neurological Implications and SARS-CoV-2’s Neuroinvasive Impact

One of the early studies on the COVID-19 patient population in Wuhan highlighted the frequent occurrence of neurological symptoms affecting muscle function and motor control [56,57,58,59]. Each hemidiaphragm is innervated by a separate phrenic nerve. Consequently, diaphragmatic palsy can result from damage to this nerve or from inherent weakness in the diaphragm muscle fibers. The causes of this diaphragmatic palsy may be neurological, traumatic, inflammatory, malignant, iatrogenic, or idiopathic [60]. Studies have demonstrated that human tissues infected with SARS-CoV-2 exhibit an increased expression of the angiotensin-converting enzyme 2 (ACE2), which potentially plays a crucial role in enabling the virus to invade not only neuronal but also muscular structures, including the diaphragm [61,62]. Given that the SARS-CoV-2 virus is neuroinvasive—a fact confirmed by the occurrence of complications such as myasthenia gravis and Guillain– Barré syndrome—diaphragm damage unrelated to mechanical ventilation is highly probable [63,64,65]. Diaphragmatic palsy can be bilateral or, more commonly, unilateral. Only a few cases of such damage have been described [65,66,67,68,69], but they are significant both clinically and in terms of further treatment.

### 3.2. Diaphragm and Inflammatory Response in COVID-19 Infection

SARS-CoV-2 is known to trigger a significant inflammatory response that affects multiple body structures. Central to this response is the ‘cytokine storm’, characterized by the uncontrolled release and activation of inflammatory cytokines and chemoattractants—molecules produced during the virus’s interaction with the host’s immune system. This cytokine activity leads to the widespread infiltration of mononuclear cells throughout various tissues, accompanied by lymphopenia. In addition to the virus’s direct impact on muscle cells, the intensified cytokine storm can further exacerbate inflammatory effects at the muscle level. As a result, an increase in C-reactive protein, a marker of inflammation, along with high levels of IL-1, IL-6, and TNF-α, triggers proteolysis in muscle fibers, promotes fibrogenesis, activates fibroblasts, and inhibits progenitor cells that are essential for the formation of new muscle fibers [70,71,72,73]. According to Shi et al. [61], histological evaluation revealed that epimysium and perimysium fibrosis was more than twice as prevalent in the diaphragms of COVID-19 patients compared to control patients in intensive care units. This phenomenon is attributed to the increased expression of genes involved in fibrosis. Studies on Duchenne muscular dystrophy indicate the important role of the cytokine IL-6 in processes related to redox balance in the diaphragm muscle. It has been shown that the excessive expression of IL-6 disrupts redox balance in the diaphragm, contributing to progressive muscle atrophy. Studies on dystrophic mice confirm that IL-6 is a key factor intensifying oxidative stress, leading to the damage of the diaphragm muscles and the worsening the course of muscular dystrophy [71,74,75,76].

It is known that the diaphragm plays a crucial role in patient prognosis due to the presence of ACE2 receptors on muscle membranes. Therefore, the diaphragm, like other skeletal muscles, may be directly at risk of damage as a result of COVID-19 infection. Moreover, SARS-CoV-2 can activate immune cells, causing both direct and indirect immune-mediated muscle impairment [76]. In relation to viral activity, it is important to consider the impact of SARS-CoV-2 on the nervous system, particularly its role in the demyelination of nerves, including the phrenic nerve. Moreover, in many cases, Guillain–Barré syndrome can occur in COVID-19 infection [77,78,79,80,81]. The fact that many patients experience muscle weakness serves as a cornerstone for diagnosis and further treatment [82].

### 3.3. Musculoskeletal Effects of COVID-19

The high incidence of skeletal muscle symptoms suggests structural and functional changes in the muscles of COVID-19 patients [83]. The recumbent position associated with hospital stays negatively impacts the musculoskeletal system, leading to muscle weakness and atrophy. It is important to note that older patients are particularly vulnerable to these changes due to the inherent sarcopenic process associated with aging [84,85,86,87]. Prolonged bed rest can significantly accelerate this process, resulting in decreased muscle mass, reduced contractile strength, increased fatigue, diminished muscle power, and deteriorated neuromuscular conduction [84,88]. Individuals with sarcopenia often face metabolic stress due to the condition’s impact on their bodies. This stress is primarily manifested through the excessive catabolism of skeletal muscles. The purpose of this catabolism is to supply crucial nutrients, specifically amino acids and glutamine, to the entire body [89,90,91]. Muscle weakness can result from decreased specific tension, muscle atrophy, or a combination of both factors. Qualitative changes in muscle tissue, such as fat infiltration and selective myosin loss, along with neuromuscular activation disorders, can contribute to reduced specific tension [92]. Diaphragmatic alterations have not been widely studied. Formenti et al. conducted an ultrasonographic evaluation of the diaphragm, rectus femoris muscle, and parasternal muscles in individuals with COVID-19-associated ARDS. The authors reported that, based on echogenicity, muscle quality was inferior in survivors compared to non-survivors. Notably, diaphragm thinning is observed not only in patients experiencing severe COVID-19 [61,93,94] but also in those with milder manifestations [95].

## 4. Exploring the Long-Term Symptoms and Conditions of COVID-19

**Figure 3:**
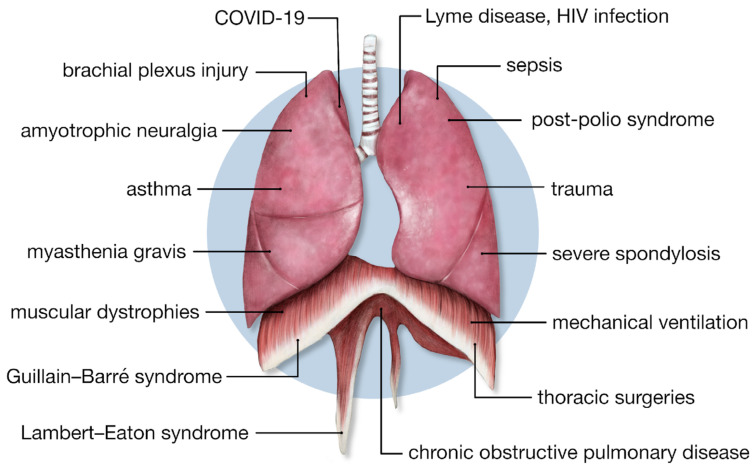
The main causes of diaphragm, vagus nerve, and lung dysfunction.

The COVID-19 pandemic has particularly highlighted the issue of the long-term effects of the disease, known as ‘long COVID’ or ‘post-COVID-syndrome’. Studies indicate that a significant number of COVID-19 patients experience musculoskeletal dysfunction, manifesting as muscle pain and general weakness in approximately 25% to 50% of cases. The most common symptoms also include dizziness, swaying, or loss of balance. Additionally, bone and joint pain frequently coexist with muscle pain, further complicating the recovery process for many individuals [7,22,88,96,97,98,99,100,101,102]. Moreover, multi-organ symptoms associated with respiratory diseases, such as the non-physiological adaptations of the diaphragm to the altered clinical situation, contribute to the complex health challenges faced by these patients [Figure 3].

Throughout the course of long COVID, various patterns of lung dysfunction have been observed, including restrictive ventilation, impaired diffusing capacity, cerebral respiratory dysregulation, and respiratory dysfunction characterized by hyperventilation. Additionally, respiratory muscle atrophy accompanied by dyspnea has been noted in recovering patients [103,104,105,106].

Studies indicate that patients with COVID-19 experience diaphragmatic dysfunction and a decrease in its thickness, suggesting atrophic changes within the muscle [7,84,94,107,108,109,110,111,112]. The change in diaphragm thickness has therefore been identified as an independent risk factor for severe COVID-19. Reduced diaphragm thickness may limit the force of the inspiratory pump, significantly accelerating the progression from mild to severe respiratory failure in the context of pneumonia. This reduction can lead to difficulty in maintaining adequate ventilation under increased inspiratory load [94,112,113,114]. The effects of diaphragmatic dysfunction can be multifaceted. In long-term observation, diaphragmatic disorders may manifest in a range of issues, from vocal disturbances to pelvic floor problems.

### 4.1. Neuromuscular Disorders and Metabolic Changes in the Course of COVID-19

Neuromuscular disorders, ranging from mild creatine kinase (CK) elevation to flaccid tetraplegia, are particularly significant in the context of diaphragmatic function [64]. The highest incidence of diaphragmatic dysfunction is observed in hospitalized post-COVID-19 patients who require inpatient rehabilitation. Farr et al. highlights the importance of critical illness myopathy, where the pathophysiology is considered multifactorial, involving muscle membrane dysfunction, microcirculatory changes during inflammation, exposure to high doses of corticosteroids, and impaired translocation of the glucose transporter type 4 to muscle membranes [115,116]. Recent reports indicate significantly reduced exercise capacity in patients with long COVID, which is associated with metabolic changes in skeletal muscles and a shift toward fibers that fatigue more quickly. Notable findings include acute exercise-induced reduction in skeletal muscle mitochondrial enzyme activity, an increased accumulation of amyloid-containing deposits in skeletal muscles, signs of severe muscle tissue damage, and a blunted T-cell response in skeletal muscles following exertion. The findings from Appelman et al. confirm that long COVID is linked to a decreased ability of skeletal muscles to perform oxidative phosphorylation, with fiber type shifts and reduced mitochondrial respiration contributing to diminished exercise capacity [117,118,119,120].

### 4.2. Voice Disorders in the Course of COVID-19

Voice problems observed in the course of COVID-19 infection, as well as in the post-COVID syndrome, are a consequence not only of respiratory system disorders, but also of impaired diaphragm activity, abnormal respiratory path, and impaired respiratory, phonatory, and articulatory coordination [121]. The latest research indicates disturbances in the quality of voice and speech, requiring speech therapy after COVID-19 infection in order to improve the general efficiency and functioning of patients in everyday life [122].

### 4.3. Pelvic Floor Muscle Disorders in COVID-19

Another clinical problem following COVID-19 is the weakness of the pelvic floor muscles. One hypothesis supporting this finding is based on the symptomatology of individuals affected by COVID-19, in which coughing is the most prevalent clinical symptom [123]. Recurring episodes of coughing may lead to fatigue in the pelvic floor muscles [124]. Additionally, the SARS-CoV-2 virus is known to use ACE2 as a host cell receptor, which is highly expressed in the small intestine and moderately expressed in the bladder [125,126]. This may contribute to the virus’s adverse effects on the gastrointestinal and urinary systems. Considering the synergistic functioning of the diaphragm with the pelvic floor muscles, multifidus muscle, and transversus abdominis muscle, the severity of respiratory dysfunction can manifest as difficulty in activating the diaphragm, increased tension or weakness of the pelvic floor muscles, lower back pain, and poor balance [127,128,129].

### 4.4. Sleep Disorders in the Course of COVID-19

Common symptoms in the course of long COVID include sleep disturbance. In addition to insomnia, SARS-CoV-2 patients frequently exhibit respiratory disorders during sleep (RDDS), the most common of which is obstructive sleep apnea (OSA) [130,131]. Diaphragmatic movement has been discussed in the literature, but there are currently no reports on the role of the diaphragm as a radiological marker of OSA. Obstructive sleep apnea is a common clinical condition characterized by repeated narrowing or collapsing of the throat during sleep, leading to obstructive sleep apnea events. The primary mechanism behind OSA is an excessive reduction in the tone of the muscles responsible for maintaining the patency of the upper airways. This reduction in muscle tone leads to repeated episodes of apnea or hypopnea due to the complete blockage or significant restriction of airflow at the throat level, despite ongoing respiratory effort. These events lead to repeated episodes of hypercapnia, hypoxia, increased fluctuations in intrathoracic pressures, and heightened sympathetic system activity [132,133]. Radiological studies have demonstrated that people with OSA exhibit greater diaphragm thickness, as measured by ultrasound, compared to healthy patients without this condition [133,134]. This may be explained by the fact that respiratory obstruction leads to increased respiratory activity, metabolic adaptations, and the hypertrophy of the respiratory muscles, which may impair their function [135]. To confirm this phenomenon, invasive tests were performed, which showed lower functional efficiency and a greater fatigue of the diaphragm in patients with severe OSA [134,136]. Although sleep disorders related to COVID-19 are more often the result of chronic stress, anxiety, and other mental disorders, the role of the diaphragm’s functioning should also be considered. At the same time, generally accepted data on the negative effects of sleep deficiency on the immune system become particularly important in the context of coronavirus infection, the pathogenesis of which requires further and deeper research [137].

## 5. Diagnosis of Diaphragmatic Dysfunction Resulting from COVID-19

The American Thoracic Society and the European Respiratory Society provide a list of tests used in assessing the respiratory system, including respiratory muscles [138]. However, diagnosing dysfunctions of the respiratory system, particularly the diaphragm in a long-term context, proves to be a challenge.

The topic of direct diaphragm damage by the coronavirus is not yet fully elucidated. Given the negative impact of COVID-19 on the respiratory system, the most critical aspects are the proper diagnosis of diaphragmatic disorders and planning for subsequent management in the event of damage. However, diagnosing diaphragmatic damage during the course of COVID-19 poses a real challenge, as standard lung function tests do not adequately reveal changes in diaphragmatic muscle strength [139,140]. The most widely available method for diagnosing diaphragmatic dysfunction is ultrasonography [141]. However, in the face of post-COVID syndrome and long-term changes, there is a high likelihood that these dysfunctions may not be revealed. Steier et al. demonstrated that in-depth respiratory muscle assessment techniques can increase the accuracy of diagnosing diaphragmatic weakness by up to 40% [142]. In the study by Regmi et al. [7], invasive techniques demonstrated that diaphragmatic weakness persisted for 15 months after coronavirus infection. Furthermore, transdiaphragmatic pressure has proven to be one of the most effective diagnostic methods from a long-term perspective. This supports the hypothesis that diaphragmatic weakness is associated with the occurrence of exertional dyspnea. Therefore, diaphragmatic weakness may explain the exertional dyspnea reported by patients in the absence of other pulmonary or cardiac abnormalities. A reduction in transdiaphragmatic systolic pressure has been observed in patients with COVID-19, similar to what is seen in patients with neuromuscular diseases and severe shortness of breath, which is important from a clinical perspective [143,144,145,146,147]. Symptoms, associated pathological findings, pathogenetic data, and prognostic outlooks, vary across different organs. This necessitates separate analytical reports for patterns affecting the lungs, cardiovascular system, neuromuscular system, and brain [148,149]. Suspected unilateral diaphragmatic paralysis, indicated by diaphragm elevation, can be demonstrated using chest X-ray or computed tomography. In acute conditions (including patients presenting with COVID-19), the diagnosis of diaphragmatic dysfunction using ultrasound can help make treatment decisions, such as ventilation and the need for ICU-level care. In the outpatient setting (including chronic COVID-19 patients), treatment options for symptomatic diaphragmatic dysfunction include noninvasive positive pressure ventilation, phrenic nerve microsurgery, and phrenic nerve or diaphragm stimulation. It is important to recognize that the regeneration of the phrenic nerve is a relatively slow process, with recovery from injury potentially taking between one and three years. Given the prevalence of respiratory complications in COVID-19 survivors, regular ultrasound examinations are highly recommended to monitor the clinical condition and guide therapeutic decisions. For patients with long-term effects from COVID-19, particularly those suffering from respiratory dysfunction, serial ultrasound examinations of the diaphragm are crucial. These examinations help track disease progression and evaluate the effectiveness of non-surgical interventions such as pulmonary rehabilitation and treatments aimed at enhancing muscle strength and function [141,150,151,152].

### Challenges in Differentiating Diaphragmatic Dysfunction

Undoubtedly, computed tomography (CT) emerges as a pivotal diagnostic tool in evaluating pulmonary disorders. It has been demonstrated that quantitative CT COVID scores at admission serve as an independent prognostic factor for predicting the rapid escalation to severe COVID-19 pneumonia. Pulmonary CT scans provide invaluable data, not only in diagnosing COVID-19 pneumonia but also in distinguishing it from other pulmonary diseases. The resolution of residual lung changes posts a negative RT-PCR test for SARS-CoV-2 can take several weeks or more [6,8]. Diaphragmatic dysfunction encompasses conditions such as eventration, weakness, diaphragmatic palsy, or diaphragmatic flutter [20]. Diaphragmatic dysfunction resulting from mechanical ventilation is a known issue, but it is challenging to differentiate this effect from the impacts of a COVID-19 infection [153]. A weakening of the respiratory muscles may also occur, for example, as a result of the dysfunction or inflammation of the phrenic nerves [154,155]. Diaphragmatic palsy involves a loss of muscle strength and may result, among other factors, from the weakening of the muscle itself or damage to its innervation. The severity of the paralysis, as well as whether it is unilateral or bilateral, determines the clinical symptoms. These symptoms can range from being unnoticeable to progressing to a condition that requires respiratory support. Symptoms resulting from diaphragm abnormalities may be confused with heart and lung pathology [156,157]. The weakness of the diaphragm may lead to a range of issues, including sleep disorders, sleep apnea syndrome, chest pain, gastroesophageal reflux, and daytime fatigue [134,158,159,160]. Additionally, diaphragmatic dysfunction is the cause of prolonged hospitalization in patients requiring assisted mechanical ventilation, oxygen therapy, or continuous positive airway pressure (CPAP) during the night [161,162].

## 6. Conclusions

This review article summarizes the current knowledge on the involvement of the diaphragm in the development of respiratory disorders resulting from SARS-CoV-2 infection. It discusses the significant impact of disorders in the nervous, endocrine, respiratory, and muscular systems on diaphragmatic function during the course of COVID-19. The critical role of the diaphragm’s thickness and strength in the progression of respiratory disorders is also highlighted. Additionally, the article focuses on new diagnostic methods that can predict the trajectory of respiratory disorders in post-COVID-19 patients. Particularly noteworthy is the use of twitch transdiaphragmatic pressure measurement, which, unlike ultrasound, has been successful in detecting the severity of diaphragmatic dysfunction in patients with long COVID-19. Table 1 presents the most significant studies on the post-COVID syndrome included in this review.

The proper diagnosis of diaphragmatic disorders during COVID-19 is crucial for selecting the appropriate treatment and predicting the risk of complications with mechanical ventilation. Testing the diaphragm through volitional maneuvers may be constrained by the patient’s ability to exert effort. Conversely, non-volitional tests that utilize neuromuscular stimulation tend to be technically intricate due to the diaphragm’s relatively inaccessible location. The awareness of the high likelihood of diaphragmatic dysfunction during COVID-19 and in the post-COVID-19 syndrome can expedite diagnostic processes and guide the direction of rehabilitation. This review article seeks to underscore that diaphragmatic dysfunctions resulting from SARS-CoV-2 infections may persist over an extended period. It is crucial to recognize that standard diagnostic techniques such as radiography or ultrasonography may not adequately reveal the multifaceted dysfunctions of the diaphragm. Special consideration should be given to pulmonary function tests, which often fail to capture underlying muscular issues, focusing instead on the functionality of the lungs alone. Muscular disorders and weaknesses arising from COVID-19, which may present as reduced grip strength, are likely to extend to the diaphragm, thereby increasing the risk of exacerbating dyspnea. This underscores the importance of comprehensive assessment and targeted rehabilitation strategies to address not only peripheral muscle dysfunction but also diaphragmatic involvement in post-COVID-19 patients. Furthermore, this review emphasizes that vocal problems observed during COVID-19, as well as in post-COVID syndrome, result from impaired diaphragmatic activity and abnormal respiratory patterns, among other factors. To the authors’ knowledge at the time of publication, this article is the first to comprehensively address diaphragm dysfunction resulting from COVID-19 and long COVID in multiple aspects. Further research is necessary on the role of the diaphragm in relation to clinical symptoms, which will enable the implementation of appropriate treatment, including comprehensive rehabilitation, speech therapy, and respiratory therapy.

## Figures and Tables

**Table 1:** The most significant studies on the post-COVID syndrome.

Topic	Reference	Conclusion
Diaphragmatic weakness continued to be evident 15 months post-hospitalization for COVID-19, even among patients who did not undergo mechanical ventilation.	Regmi et al. [7]	Transdiaphragmatic pressure has been demonstrated to be one of the most effective long-term diagnostic methods.
During the course of long COVID, several patterns of lung dysfunction have emerged, including restrictive ventilation, reduced diffusing capacity, cerebral respiratory dysregulation, and respiratory dysfunction characterized by hyperventilation.	Han et al. [104] van Wincoop et al. [105] Faverio et al. [106]	Atrophy of the respiratory muscles, accompanied by dyspnea, has been observed in patients who are recovering.
One year after severe COVID-19 ARDS, diaphragmatic dysfunction with impaired activation may persist and contribute to exertional dyspnea.	Spiesshoefer et al. [107]	The function of the diaphragm and its voluntary central activation are potential correlates with exertional dyspnea following acute respiratory distress syndrome (ARDS).
Dysphonia may occur due to coronavirus infection.	Gacka [121,122]	Voice issues during COVID-19 stem from respiratory system disorders, diminished diaphragm activity, abnormal respiratory pathways, and compromised respiratory, phonatory, and articulatory coordination.

## Data Availability

No new data were created or analyzed in this study. Data sharing is not applicable to this article.
